# Preliminary Efficacy of a Cognitive Behavioral Therapy–Based Smartphone App for Smoking Cessation in China: Randomized Controlled Pilot Trial

**DOI:** 10.2196/48050

**Published:** 2024-03-18

**Authors:** Shanshan Chen, Jinsong Tang, Congyang Wu, Ge Zhang, Jing Zhang, Yanhui Liao

**Affiliations:** 1 Sir Run Run Shaw Hospital Zhejiang University School of Medicine Hangzhou China; 2 Johnson & Johnson Pharmaceutical Company Shanghai China

**Keywords:** China, cognitive behavioral therapy, program acceptability, randomized controlled trial, smartphone app, smoking cessation

## Abstract

**Background:**

The overall prevalence of cigarette smokers in China is very high, and China’s total cigarette consumption makes up more than 40% of the world’s consumption. In view of the lack of smoking cessation services and social support in China and the effectiveness of mobile phone apps for quitting smoking in other countries, we carried out a smartphone app–based smoking cessation trial in China.

**Objective:**

This study aimed to evaluate the efficacy of a cognitive behavioral therapy (CBT)–based smoking cessation smartphone app among smokers seeking treatment in China.

**Methods:**

We conducted a randomized controlled, web-based pilot clinical trial in China between February 23 and June 27, 2021. Eligible participants were randomly assigned to the smoking cessation app intervention group or the control group in a ratio of 1:1. The intervention group received the CBT smoking cessation intervention using a smartphone app, and the control group received a “thank you” message. The intervention was 4 weeks long, and the patients were followed up for 4 weeks. The primary outcome was self-reported continuous smoking abstinence at week 4 after the quit date. The secondary outcomes included self-reported 7-day point prevalence of smoking abstinence; reduction of the number of cigarettes smoked per day at weeks 1, 2, 3, and 4; and program acceptability.

**Results:**

A total of 973 people were recruited to quit smoking, of whom 262 completed basic information, 56 were excluded, and 206 were randomized and included in the final analysis. There were 189 (91.7%) men and 17 (8.3%) women, with an average age of 34.46 (SD 7.53) years and an average daily smoking rate of 15.93 (SD 7.10) cigarettes/day. We found 30 (29.7%) of the 101 participants in the intervention group and 7 (6.7%) of the 105 participants in the control group reported continuous smoking cessation after the quit date at week 4 (odds ratio 5.92, 95% CI 3.78-9.26; *P*<.001). The 7-day point prevalence abstinence rate of the intervention group varied from 42.6% (43/101) to 46.5% (47/101) after 1, 2, 3, and 4 weeks, while the control group varied from 18.1% (19/105) to 26.7% (28/105). Compared to the control group, continued smokers consumed 1.5-3.0 fewer cigarettes per day in the intervention group. The overall program got positive user feedback with a high satisfaction rate (66/87, 76%) and an average Mobile Application Rating Scale user version score of 3.46.

**Conclusions:**

Our pilot study provided preliminary evidence that the CBT-based smoking cessation smartphone app led to improved smoking quit rates versus control in Chinese smokers. The study demonstrated the CBT-based smartphone app may be an effective and feasible digital treatment model to help smokers quit, which may improve smoking cessation service quality and accessibility in China.

**Trial Registration:**

ClinicalTrials.gov NCT04421170; https://clinicaltrials.gov/study/NCT04421170

**International Registered Report Identifier (IRRID):**

RR2-10.1136/bmjopen-2020-041985

## Introduction

Tobacco smoking remains one of the leading causes of preventable death [1]. More than 8 million smokers worldwide die each year from smoking-related causes, of whom about 7 million die of diseases caused by smoking and about 1.2 million die from diseases caused by secondhand smoke exposure [2]. More than 1 million people in China lose their lives due to smoking every year. If effective action were not taken to significantly reduce the smoking rate, the number of deaths due to smoking would increase to 2 million per year by 2030 and 3 million per year by 2050 [3]. Since 1978, cigarette production in China has grown from 20% to over 40% of the world’s cigarette products [4]. Tobacco has caused a huge economic burden to society and individuals, including the high cost of medical care, more diseases, and premature death [5]. According to survey results on adult smoking prevalence in China in 2018, the smoking rate of people aged 15 years or older in China was 26.6% [6], which was still higher than in many other countries.

One of the key reasons for China’s low smoking cessation rate is limited smoking cessation services. The 2018 China Adult Tobacco Survey demonstrated that 90.1% of smokers who tried to quit in the past 12 months did not use any form of cessation aid [6]. A report in 2019 showed that 366 hospitals and primary health care institutions in China set up smoking cessation clinics, of which only 43% provided smoking cessation medications [7]. Few smokers were willing to go to the smoking cessation clinic for help [5], and most self-help cessation attempts failed [8,9]. Most smokers tend to relapse in the first few weeks after trying to quit smoking [10]. The success rate of quitting smoking after 1 year was 3%-5% for those without support, 7%-16% when smokers received behavioral intervention, and as high as 24% when smokers received drug treatment and behavioral help [11]. At present, it is necessary and meaningful to find accessible, effective, and scalable smoking cessation intervention methods to improve smoking cessation services in China.

Nowadays, mobile phone–based smoking cessation support provides a new channel for those who cannot get access to or lack the willingness to use face-to-face support [12]. About 45% of mobile phone subscriptions worldwide were related to smartphones, and this number continues to grow as 75% of new mobile phone sales were smartphones [13]. Around the world, mobile phones are becoming more and more useful in health information and health care service delivery [14]. Digital medical service has the advantages of economy, easy access [12], and easy promotion, which provides an opportunity for developing cost-effective smoking cessation digital interventions [15]. We previously carried out a study on smoking cessation intervention based on SMS text messaging (Happy Quit) in China, which supported the effectiveness and feasibility of digital medical services [16]. Smartphone apps for health and health care are increasing rapidly, but there are few in the smoking cessation area [17]. Smartphone apps can enable diverse functions, including audio and video materials, and can provide additional resources through the network [18]. Besides, smartphone apps could be more powerful than SMS text messaging programs for digital intervention because they have the potential to increase user engagement through diversified user interfaces and user experiences [19]. A study has found that under the same intervention content, the effect of a smartphone-based smoking cessation intervention app was stronger than that of a non–mobile device–based web page intervention. Mobile devices have the potential to make it easier for smokers to get smoking cessation support [20]. However, most of the currently available smartphone smoking cessation apps on the market have the problem of low compliance with standard clinical practice guidelines [21]. In 2018, a study of smoking cessation apps for the UK mobile phone app market found that most smoking cessation apps had low theoretical adherence, and the overall quality of smoking cessation apps was still unsatisfactory [22]. A review showed that there was insufficient evidence of the smartphone app’s effectiveness on smoking cessation support; thus, more randomized controlled trials (RCTs) were recommended to validate the smartphone-based digital smoking cessation interventions [12]. Specifically, as far as we know, there has been no mobile smoking cessation app with sufficient clinical evidence in China.

As an indispensable part of psychotherapy, cognitive behavioral therapy (CBT) plays an important role in the field of psychological and behavioral intervention [23]. One of the roles of CBT involves challenging behaviors that may trigger or sustain difficulties, such as smoking. CBT has been proven to help reduce cravings and promote smoking cessation by changing participants’ thoughts and behaviors [24]. The CBT quitting method consists of several parts, generally including preparation before quitting smoking, starting quitting smoking, and maintaining or preventing the recurrence treatment stage [25]. CBT may be a good choice for people who want to quit smoking, especially for those who want to quit smoking through nondrug methods [25,26]. Our previous study on “Happy Quit” SMS text message smoking cessation in China found that a CBT-based smoking cessation intervention can effectively improve the 24-week continuous smoking cessation rate [16,27]. As far as we know, there is little research to explore the effect of CBT-based mobile apps on smoking cessation intervention. Therefore, we are committed to designing a CBT-based mobile app for Chinese smokers who want to quit smoking, so as to find more effective ways to quit smoking.

Considering the above factors, in order to provide more feasible and effective smoking cessation services, we developed a scientific mobile smoking cessation app based on clinical practice guidelines. Smoking is an unhealthy behavior that can be altered, and CBT can help solve a wide range of smoking cessation problems. Various forms of interventions were developed in the app based on CBT to help smokers learn new skills, resist smoking cravings [28], and better deal with emotional disorders [29].

This Mandarin mobile smoking cessation app, based on CBT, integrates smoking cessation support and social skills training and finally achieves the goal of cognitive behavior change. A previous 1-arm study on the feasibility and acceptability of this CBT-based smartphone app was carried out for Chinese smokers who wanted to quit and showed that the smoking cessation app may become a new digital therapy model and have the potential to provide support for smoking cessation services in China [30]. We hypothesized in this study that the CBT-based smoking cessation app is feasible and acceptable and can significantly increase the quit rate.

## Methods

### Objectives

In the current trial, the objectives were to evaluate the feasibility and acceptability of this Chinese CBT-based app in a direct-to-participant clinical design and preliminarily evaluate the efficacy of the CBT-based app for smoking cessation in China. Given that CBT [23] is the current standard in behavioral intervention for smoking cessation, we tested the hypotheses that participation in this intervention will lead to significant improvement in the self-reported continuous smoking cessation rate at 4 weeks; self-reported 7-day point prevalence smoking abstinence and reduction of the number of cigarettes smoked per day at weeks 1, 2, 3, and 4; and that the program would be acceptable to participants.

### Study Design

This was a randomized controlled, direct-to-participant clinical trial conducted in China. Researchers conducted preliminary conditional screenings for each participant. In the baseline assessment, all eligible participants were required to fill out a baseline questionnaire that included demographic information, motivation to quit smoking and willingness.

**Figure 1:**
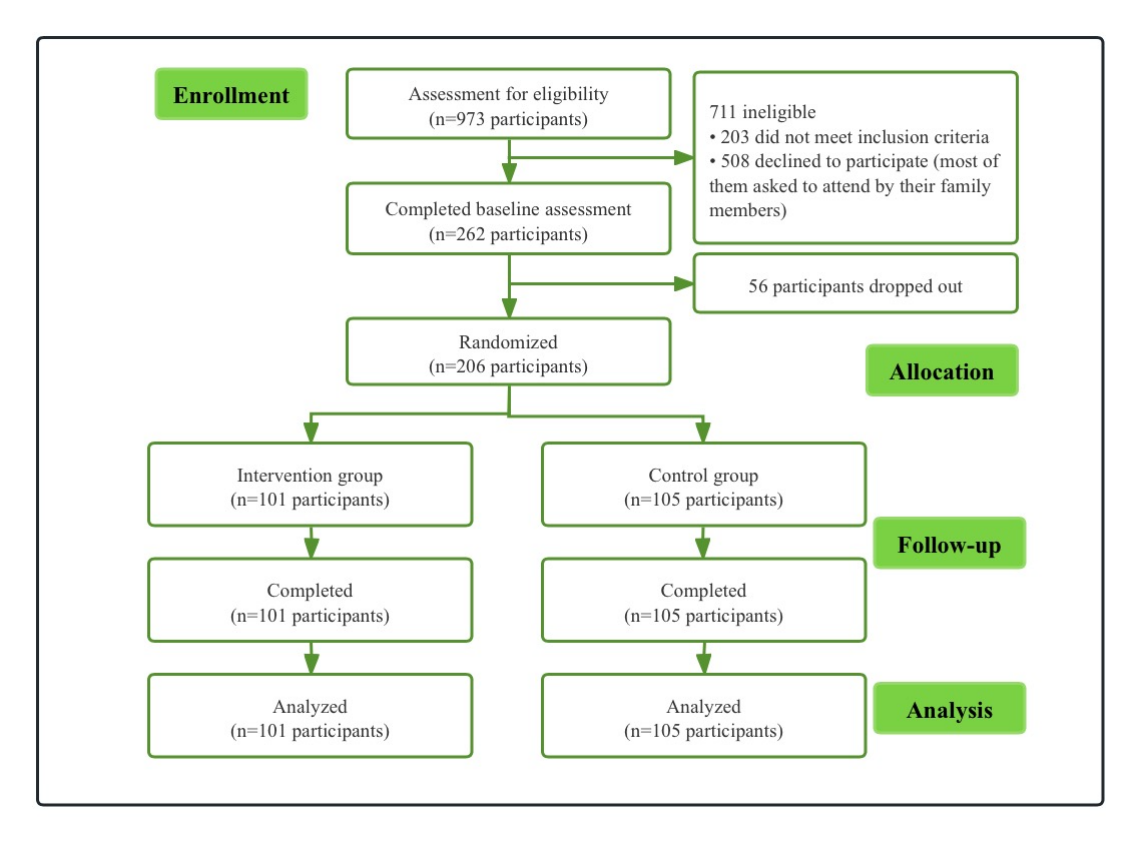
Flowchart of the study.

Using a randomization method by electronic data capture system, participants were randomly assigned to a smoking cessation app intervention group or to a control group in a 1:1 ratio after the completion of the screening, consent, and baseline questionnaires. No changes have been made to the trial design since its commencement. Participants in the intervention group received the CBT-based smoking cessation app, while those in the control group were encouraged to quit but were not provided with the CBT-based smoking cessation app. A control group was used since this was an exploratory study designed to assess both the feasibility, acceptability, and initial efficacy of the app intervention. Participants were required to complete the program acceptability assessment, including the App Satisfaction Assessment Scale and the Mobile Application Rating Scale, user version (uMARS; including engagement, functionality, aesthetics, information, and the average score of the uMARS total score), 4 weeks after the quit date. Each item is scored from 1 to 5, with a maximum total score of 5.0. The higher the score, the better the satisfaction [31]. Follow-up visits were conducted at weeks 1, 2, 3, and 4 after the participants started smoking cessation. The study design is depicted in Figure 1.

### Participants

The eligibility criteria for participants are shown in Textbox 1.

**Eligibility criteria for participants.**

**Inclusion criteria**

- Cigarette smokers (smoked more than 100 cigarettes in their lifetime and currently smoke 5 or more cigarettes a day)
- Aged 25 years or older
- Able to read and write in Chinese
- Owning a smartphone (operating system: iOS or Android)
- Having experience in using apps
- Expressing an interest in quitting smoking within the next month
- Willing to provide informed consent to participate in the study
- Able to follow up for at least 1 week

**Exclusion criteria**

- Nonsmokers or only use electronic cigarettes
- Smokers without previous “serious” attempts to quit smoking (Motivation to Stop Scale score <7)
- Currently experiencing severe mental illness
- Had already started their quit attempt or used any smoking cessation treatment at the time of registration
- Unable to use smartphones and apps
- Did not have sufficient command of Chinese to participate in the trial

### Procedures

#### Sample Size

A sample size of about 200 participants was calculated to give 80% power and a 2-sided 5% significance for detecting a beneficial difference in the self-reported continuous smoking cessation rates between the CBT app intervention and the control intervention. These assumptions were made based on our previous RCT of CBT-based SMS text messaging interventions for smoking cessation [16] and other previous RCTs of smartphone app interventions for smoking cessation [15], as well as the consideration of the better efficacy of the CBT-based app than the non–CBT-based app for smoking cessation [32]. A total of 200 participants randomized 1:1 to each arm (n=100) would obtain a 95% CI width estimate of ±10% for the CBT-based app for a 4-week self-reported continuous quit rate. The 20%-30% CBT-based app quit rate CI (95% CI) was designed to provide precision in estimating the main study of CBT-based app intervention. There were no planned interim analyses or stopping rules.

#### Recruitment

From February 23 to June 27, 2021, a total of 206 participants were recruited through advertisements on social media, such as WeChat (Tencent), Weibo, the website, and the principal investigator–affiliated hospital WeChat official account. Potential participants were screened through a mobile phone or WeChat phone contact. Of the 973 screened, 711 did not meet the preliminary inclusion and exclusion criteria. Of the 262 baseline assessments completed, 56 were deemed ineligible to participate. Therefore, a total of 206 participants were randomized and included in the analysis according to the intention-to-treat (ITT) principle.

Recruitment was undertaken through (1) the telephone number for quitting smoking and (2) WeChat, a popular social media in China. For recruitment through the telephone number, research investigators directly contacted these participants about the study by phone call. For recruitment through WeChat, participants were invited to make a WeChat voice or video call to research investigators. A link to informed written consent was sent by email to each eligible participant, and e-consent was obtained from all participants before the study commencement and data collection.

#### Stratified Block Randomization

Randomization was conducted by an electronic data capture system, a computerized system designed for the collection of clinical data in electronic format for RCTs and other clinical trials. Randomization was at the level of the individual participant with a 1:1 ratio by the method of minimization stratified by balancing for (1) with or without previous quit attempts and (2) 10 cigarettes or more per day. The study investigators were blinded to participants’ treatment allocation until all data were collected. The investigators who analyzed the data were also blinded to participants’ allocated groups until analysis completion.

### Interventions

#### Overview

Following randomization, participants set a smoking cessation day. On that day, participants began receiving personalized messages to help quit smoking through either a smartphone app (intervention group) or by simply receiving SMS text messages (control group). The intervention group also logged into the smoking cessation app, started a smoking cessation journey, completed tasks, and sought personalized smoking cessation assistance under the automatic guidance of the app, while the control group received regular SMS text messages.

#### Control Group

Participants in the control group received information to thank them for their participation and to remind them to complete their smoking status at each point.

#### Intervention Group

Participants from the intervention group were invited to download the CBT-based app. The app integrates cognitive-behavioral principles and tailored behavior-change skills in Chinese. More descriptive details of this smartphone app were available elsewhere [33]. Participants who were randomly assigned to the intervention group were instructed on how to access and use the app, including a schedule of intervention activities, tasks during the pre- and postpreparation stages, and expected completion dates. Participants could begin their assigned tasks immediately after completing the pretest assessment. The system limited participants to 3 tasks each day during the preparation stage. To encourage completion, SMS text message reminders were sent each week after the quit date.

#### Both Groups

All participants earned CNY 20 (US $2.8) phone top-ups for the completion of pre- and posttest questionnaires. Participants from both groups received a reminder by SMS text message to complete questions about their smoking status during weeks 1, 2, 3, and 4 after the quit date by electronic Patient Reported Outcomes (ePRO) software.

### Outcome Measures

#### Participant Demographics and Smoking Behaviors at Baseline

Participant demographic and smoking behaviors at baseline, including gender, age, height, weight, education, cigarettes per day, quit history, motivation, self-efficacy to quit, quitting smoking gradually or abruptly, smoking craving, and severity of nicotine dependence, were evaluated using the Fagerstrom Test for Nicotine Dependence (FTND).

#### 4-Week Continuous Smoking Abstinence

Continuous smoking abstinence for 4 weeks is defined as smoking no more than 5 cigarettes in the past 4 weeks since quitting, measured through response to the following item: (1) “How many cigarettes have you smoked in the last 4 weeks?” Response choices were “0,” “1-5,” or “more than 5”; and (2) “If ‘more than 5,’ recorded the number of smoked cigarettes per week.” Within 4 weeks, participants who smoked more than 5 cigarettes indicated a recurrence [34]. This was the primary outcome measure.

#### 7-Day Point Prevalence Smoking Abstinence

Self-reported abstinence of at least 7 days before the assessment day was assessed 1, 2, 3, and 4 weeks after each participant’s quit date. It is defined as smoking no more than 5 cigarettes in the past 1, 2, 3, and 4 weeks since quitting.

#### Reduction of the Number of Cigarettes Smoked Per Day

Daily cigarette consumption among participants who were still smoking after quitting days (participants who smoked more than 5 cigarettes in total but less than 1 cigarette per day were considered to have smoked 1 cigarette per day).

#### Program Acceptability

A questionnaire of program acceptability was assessed at 4 weeks post quitting, including the App Satisfaction Assessment Scale and the uMARS. Questions are shown in the Results section.

### Safety

The safety of this program was evaluated by the collection and analysis of spontaneous adverse events reported by participants. No serious adverse events were reported by participants in our trial.

### Intervention Effects

The primary outcome was the self-reported continuous smoking abstinence rates at 4 weeks after the quit date. Secondary outcomes included self-reported 7-day point prevalence smoking abstinence at weeks 1, 2, 3, and 4, reduction of the number of cigarettes smoked per day from week 1 to week 4, program acceptability, and the association of outcomes with baseline data. In all analyses, participants who dropped out or were lost to follow-up were considered treatment failures and smokers. Participants using smoking cessation methods not allowed in this study were not included in the efficacy analysis.

### Statistical Analysis

Statistical analyses were conducted with R software (R Foundation for Statistical Computing) and SPSS (version 23; IBM Corp). All baseline data comparisons, including participant demographics and smoking behaviors, were conducted using a chi-square test. A total of 206 participants were included in the analysis according to the ITT principle. In both groups, nominal and ordinal demographics were compared using chi-square tests, and continuous variables were compared using independent samples 2-tailed *t* tests. The number of average cigarettes smoked per day was compared using a 2-sample *t* test between groups. The mixed-effects model was used to test the self-reported smoking abstinence rates in intervention groups and control groups. Program acceptability (treatment satisfaction ratings) for participants with the app intervention was evaluated by tallying the proportion of users answering each item on the usability measure. If the smoking status was not available after quitting, the participant was considered to have smoked the same number of cigarettes per day as before quitting. The odds ratio was used as a measure of outcomes in the intervention group compared to the control group. Multiple imputations will be used in chained equations. A 2-sided *P*<.05 was used to determine statistical significance.

### Ethical Considerations

The study protocol was approved by the ethics committee of Sir Run Run Shaw Hospital, an affiliate of Zhejiang University School of Medicine (protocol number 20200129-33), and was published [33]. The trial was performed in accordance with the Declaration of Helsinki. Informed consent was obtained from all participants. The purpose, procedures and measurements, potential risks, and benefits of the trial were explained to each participant before recruitment. The investigators who analyzed the data were also blinded to participants’ allocated groups until analysis completion.

## Results

### Follow-Up Rate

Figure 1 shows the process of screening, grouping, and follow-up of participants in this study. A total of 206 participants were recruited from February 23 to June 27, 2021. Of the 973 screened, 711 did not meet the preliminary inclusion and exclusion criteria. Overall, 262 completed baseline assessments, but 56 were deemed ineligible to participate. Therefore, a total of 206 participants were included in the analysis according to the ITT principle.

### Participant Characteristics

Demographics and smoking characteristics at baseline for all participants are presented in Table 1. There were no statistically significant differences in the baseline characteristics of the 2 groups of participants (*P*>.05). A total of 206 participants were enrolled in the trial. There were 189 (91.7%) men and 17 (8.3%) women, with an average age of 34.46 (SD 7.53) years and an average daily smoking rate of 15.93 (SD 7.10) cigarettes per day.

**Table 1:** Baseline characteristics of study groups.

Characteristic		Intervention (n=101)	Control (n=105)
**Gender, n (%)**			
	Man	94 (93.1)	95 (90.5)
	Woman	7 (6.9)	10 (9.5)
Age (years), mean (SD)		34.62 (8.03)	34.30 (7.04)
**Age (years), n (%)**			
	25-34	58 (57.4)	61 (58.1)
	>34	43 (42.6)	44 (41.9)
**Education (years), n (%)**			
	≤12	14 (13.9)	12 (11.4)
	>12	87 (86.1)	93 (88.6)
Number of cigarettes smoked per day, mean (SD)		15.96 (7.46)	15.90 (6.77)
**Cigarettes smoked per day, n (%)**			
	≤10	39 (38.6)	38 (36.2)
	11-20	49 (48.5)	55 (52.4)
	21-30	10 (9.9)	11 (10.5)
	>30	3 (3)	1 (1)
Number of previous quit attempts, mean (SD)		2.07 (5.47)	3.33 (13.7)
**Previous quit attempts, n (%)**			
	Never	35 (34.7)	36 (34.3)
	1-5 times	62 (62.4)	64 (61)
	≥6 times	4 (4)	5 (4.8)
FTND^a^ score, mean (SD)		3.62 (2.40)	3.62 (2.19)
**FTND score**			
	<4 (low dependence)	55 (54.5)	55 (52.4)
	4-6 (moderate dependence)	29 (28.7)	37 (35.2)
	>6 (high dependence)	17 (16.8)	13 (12.4)
BMI, mean (SD)		24.39 (2.96)	24.54 (3.91)
Smoking (years), mean (SD)		13.85 (7.88)	14.50 (6.65)
Motivation to quit, mean (SD)		9.04 (1.48)	8.94 (1.31)
Self-efficacy to quit, mean (SD)		8.03 (2.37)	8.13 (1.89)
VAS^b^ score, mean (SD)		6.77 (2.53)	6.81 (2.16)

^a^FTND: Fagerstrom Test for Nicotine Dependence.

^b^VAS: Visual Analogue Scale (VAS measures the degree of craving for smoking; 0-10 points, where 0 represents no craving and 10 represents strong craving.

### The Outcome of Abstinence Rates

#### The Primary Outcome

Compared with 7/105 (6.7%) participants in the control group, self-reported continuous smoking abstinence at week 4 was higher (30/101, 29.7% participants) in the intervention group (odds ratio 5.92, 95% CI 3.78-9.26; *P*<.001; Table 2).

**Table 2:** Primary and secondary outcomes.

Outcome				Intervention participants (n=101), n (%)		Control participants (n=105), n (%)	OR^a^ (95% CI)		*P* value^b^
**Primary outcome**									
	Self-reported continuous abstinence at 4 weeks		30 (29.7)		7 (6.7)		5.92 (3.78-9.26)	<.001	
**Secondary outcomes**									
	**Self-reported 7-day point prevalence of abstinence**								
		1 week	47 (46.5)		28 (26.7)		2.39 (1.78-3.22)	.003	
		2 weeks	43 (42.6)		19 (18.1)		3.36 (2.43-4.64)	<.001	
		3 weeks	43 (42.6)		21 (20)		2.97 (2.16-4.07)	<.001	
		4 weeks	45 (44.6)		20 (19)		3.42 (2.48-4.70)	<.001	

^a^OR: odds ratio.

^b^Bonferroni corrected *P* values.

#### The Secondary Outcomes

Table 2 details the results of self-reported 7-day point prevalence of smoking abstinence at weeks 1, 2, 3, and 4. It was shown that the self-reported 7-day point prevalence of smoking abstinence at weeks 1, 2, 3, and 4 of the intervention group were 46.5% (47/101), 42.6% (43/101), 42.6% (43/101), and 44.6% (45/101), respectively, while 7-day point prevalence of smoking abstinence in the control group was 26.7% (28/105), 18.1% (19/105), 20% (21/105), and 19% (20/105), respectively. The differences were statistically significant at all time points. Compared to the control group, continued smokers consumed 1.5-3.0 fewer cigarettes per day in the intervention group (Table 3).

**Table 3:** Cigarettes consumed per day in nonabstinent participants (only those who self-reported smoking more than 5 cigarettes [reaching the standard of relapse] after the quit date were included).

Assessment point		Intervention group		Control group		*P* value
		Participants, n	Mean (SD)	Participants, n	Mean (SD)	
Baseline		101	15.96 (7.46)	105	15.90 (6.77)	.45
**Follow-up**						
	1 week	54	8.81 (5.56)	77	10.70 (7.06)	.10
	2 week	64	9.53 (5.69)	94	10.98 (7.57)	.22
	3 week	68	9.21 (5.58)	95	11.29 (6.85)	.06
	4 week	71	9.50 (5.58)	98	11.67 (8.17)	.09

### The Acceptability and Feasibility of the CBT-Based Smoking Cessation App Program

Participants were required to complete the program acceptability assessment, including the App Satisfaction Assessment Scale and uMARS, 4 weeks after their quit date. All participants (n=101) in the intervention group were contacted to evaluate the program based on their experience, and we finally obtained responses from 87 people, including 87 on the App Satisfaction Assessment Scale and 84 on the uMARS. The overall program satisfaction was 76% (66/87) and the percentage of dislikes was less than 10% (9/87). The uMARS included the average scores of 5 items scored 1-5, namely, engagement (2.96), functionality (3.73), aesthetics (3.56), information (3.6), and the average score of the uMARS total score (3.46). More details are available in Tables S1 in Multimedia Appendix 1 and Table 4.

**Table 4:** Descriptive analysis of Mobile Application Rating Scale user version subdomains total score (n=84).

	Mean (SD)	Median (IQR)	Minimum	Maximum
Engagement	2.96 (0.76)	3.00 (2.60-3.40)	1.00	5.00
Functionality	3.73 (0.81)	3.75 (3.25-4.25)	1.50	5.00
Aesthetics	3.56 (0.81)	3.67 (3.00-4.00)	1.00	5.00
Information	3.60 (0.92)	3.75 (3.00-4.25)	1.00	5.00
Average of total score^a^	3.46 (0.71)	3.47 (3.06-3.92)	1.53	5.00

^a^Average of total score = total score (engagement + functionality + aesthetics + information)/4.

## Discussion

### Principal Findings

In this study, our main results indicate that (1) the 4-week continuous smoking cessation rate of the intervention group participants (30/101, 29.7%) was statistically significantly higher than that of control group participants (7/105, 6.7%); (2) the self-reported 7-day point prevalence smoking abstinence rate in the intervention group at the weeks 1, 2, 3, and 4 was higher than that in the control group (43/101, 42.6% to 47/101, 46.5% vs 19/105, 18.1% to 28/105, 26.7%); (3) compared with the control group, those who continued to smoke in the intervention group smoked 1.5-3.0 fewer cigarettes a day; (4) the app had high overall satisfaction rating (66/87, 76%) and average uMARS scores (3.46). These findings suggest that the CBT-based smartphone app is effective for smoking cessation, and it could be an effective, feasible, and easy-to-access quitting app in China. It is hopeful for this app to be introduced to a large-scale Chinese population to intervene in smoking cessation.

The effectiveness of this CBT-based smartphone app intervention on short-term smoking cessation rates was encouraging. About half (47/101, 46.5%) of participants in the intervention group and about a quarter (28/105, 26.7%) in the control group reported 7-day abstinence in the first week after quitting, which was comparable to the preliminary results of other randomized controlled digital intervention studies on smoking cessation [35,36]. Previous research results for smartphone apps and SMS text messaging smoking cessation programs were similar to ours [16,37]. Participants in the intervention group reported point prevalence that was 3 to 4 times higher than that in the control group from 1 to 4 weeks, and self-reported continuous abstinence at 4 weeks in the intervention group was similar to that in previous smartphone smoking cessation app studies [37,38].

The results of this study show that the smoking cessation rate is high, and there have been some similar or inconsistent studies in the past, although there are some differences in smoking cessation standards. A systematic evaluation of smartphone apps for quitting smoking found that the results of 11 RCTs were not consistent. Among these 11 studies, the abstinence rate of the test group was significantly higher than that of the control group in 4 studies, the abstinence rate of the test group was not significantly higher than that of the control group in 5 studies, and there was no difference between the test and control groups in 2 studies [1]. The social environment in China is still quite tolerant of smokers and appears less supportive of quitting behavior, so potential quitters in China may be more resistant to personal intervention than smokers in other countries [16]. As far as we know, there are no mobile smoking cessation platforms developed under the theoretical guidance of professional medical institutions in the domestic software development market, and most of the smoking cessation apps in China are developed by individuals or companies that may not have knowledge of clinical smoking cessation practice. Those apps comply less with the recommendations from the China clinical smoking cessation guidelines (2015 edition), and their smoking cessation effect is limited [21]. Therefore, for most Chinese smokers, using apps to quit smoking is a new way to quit smoking that needs further improvement.

China has introduced some smoking control measures, but the choice of a mobile smoking cessation service is relatively limited. The earliest method of smoking cessation based on mobile phones was mainly SMS text messages [39]. Our team previously carried out an SMS text message smoking cessation project, and the effect of smoking cessation was relatively significant [16]. China’s smoking cessation services are inadequate. An authoritative national survey found that in 31 provinces, only 366 hospitals and primary care centers offered smoking cessation clinics [7]. Among them, smoking cessation clinics are mostly attached to tertiary or secondary hospitals, but population-level interventions rely heavily on primary care settings [40], where fewer clinics exist. In addition, the limited use of smoking cessation medications in China is also a serious problem [7]. Additionally, many smokers who want to quit smoking have low awareness of and use of smoking cessation support services [41].

It is noteworthy that the overall program satisfaction (66/87, 76%) and the average score of the uMARS total score (3.46) were high. About 80% (69/87) of the participants would happily recommend the app to others, which was consistent with the results of a randomized clinical trial of a smartphone app based on acceptance and commitment therapy for quitting smoking [37]. In this study, except for engagement, the software’s functionality, aesthetics, information, and the average score of the uMARS total score were all around 3.5, indicating that the participant’s overall satisfaction level was relatively high. Similar to our results, a study showed that participants made suggestions, including making features more ramified and integrating with some social media platforms to increase app and user interactivity, although some smartphone smoking cessation apps were widely accepted [41].

This pilot study provided valuable learning for future research. A review study showed that the certainty of evidence comparing smartphone apps to very low-intensity smoking cessation support was very low, and more RCTs were needed to test these interventions [12]. Also, the recruitment of participants in the intervention and control groups of RCTs related to smartphone apps for smoking cessation was not easily supported [41]. It is worth mentioning that we recruited eligible participants in this preliminary study and carried out RCTs in accordance with the norms, obtaining relatively accurate data and also providing a reference and basis for subsequent RCT studies. These preliminary data still provide sufficient and reliable information for our subsequent larger, robust trials.

### Limitations and Strengths

There are some limitations to our study. First, this study was a pilot study with a small sample size, and the results may be biased. At present, our large-sample trial has begun, which will help to further confirm our preliminary research results. Second, due to the need to master the basic ability to use the app, the enrolled smokers were relatively younger and had a higher education level, so the smoking cessation effect of the smoking cessation APP is uncertain for older or less educated smokers, and more caution needs to be exercised if the results are generalized to older or less educated smokers. Finally, some important subgroup analyses, such as smoking cessation rates between men and women, tobacco users, and e-cigarette users, were not considered in this study. Although we matched the 2 groups of quitters on factors such as gender and smoking status, these differences could potentially have an impact on smoking cessation outcomes. Despite these limitations, this study’s strengths included its conduct in China, the direct-to-participant design, and the use of an RCT.

### Conclusions

This pilot study of a CBT-based smartphone smoking cessation app provided preliminary evidence that the app led to improved smoking cessation rates versus control in Chinese smokers. This study demonstrated that the CBT-based smartphone app may be an effective and feasible digital treatment model to quit smoking, which may improve smoking cessation services in China.

## Appendix

Multimedia Appendix 1 Questions for assessing program satisfaction.

Multimedia Appendix 2 CONSORT-eHEALTH checklist (V 1.6.1).

## Abbreviations

CBT: cognitive behavioral therapy
ePRO: electronic Patient Reported Outcomes
FTND: Fagerstrom Test for Nicotine Dependence
ITT: intention to treat
RCT: randomized controlled trial
uMARS: Mobile Application Rating Scale user version

## Data Availability

All data in this study will be available from the corresponding author on reasonable request and upon completion of the data user agreement.
