# Supplementary material for: Preliminary Efficacy of a Cognitive Behavioral Therapy–Based Smartphone App for Smoking Cessation in China: Randomized Controlled Pilot Trial
Source: JMIR Form Res. 2024 Mar 18;8:e48050. doi: 10.2196/48050 (PMC10985609; doi:10.2196/48050)
Supplement: Multimedia Appendix 1 [file formative_v8i1e48050_app1.docx]

**Table S1.** Questions for assessing “cognitive behavioral therapy–based smoking cessation app” program satisfaction.

| **Question** | n^c^=87 | n=87 | n=87 | n=87 | n=87 | n=87 | n=87 |
| --- | --- | --- | --- | --- | --- | --- | --- |
|  | **Like very much+ Like somewhat** | **Like very much** | **Like somewhat** | **Neutral** | **Dislike somewhat** | **Very dislike** | **Dislike somewhat+ Very dislike** |
| Overall rating of the program | 66 (75.9%) | 22 (25.3%) | 44 (50.6%) | 13 (14.9%) | 6 (6.9%) | 2 (2.3%) | 8  (9.2%) |
|  | **Very likely+ Somewhat likely** | **Very likely** | **Somewhat likely** | **Neutral** | **Unlikely** | **Not at all likely** | **Unlikely+ Not at all likely** |
| Appraisal of “CBT-based smoking cessation app” program: Likelihood of recommending program to others | 69 (79.3 %) | 22 (25.3 %) | 47 (54.0%) | 13 (14.9%) | 1 (1.1%) | 4 (4.6%) | 5  (5.7%) |
|  | **Strongly agree+ Agree** | **Strongly agree** | **Agree** | **Neutral** | **Disagree** | **Strongly disagree** | **Disagree+ Strongly disagree** |
| The experience of using app: ease-of-use | 55 (63.2 %) | 20  (23. 0%) | 35 (40.2%) | 27 (31.0 %) | 3  (3.4 %) | 2  (2.3 %) | 5  (5.7 %) |
| The experience of using app: promoting engagement | 56 (64.3 %) | 17 (19.5 %) | 39 (44.8%) | 24 (27.6 %) | 5  (5.7 %) | 2  (2.3 %) | 7  (8.0 %) |
| The program made it easier to quit smoking | 56 (64.3 %) | 17 (19.5 %) | 39 (44.8%) | 25 (28.7 %) | 4  (4.6 %) | 2  (2.3 %) | 6  (6.9 %) |
| The program is not the one I was expected | 32 (36.8 %) | 14 (16.1 %) | 18 (20.7%) | 38 (43.7%) | 14 (16.1%) | 3  (3.4%) | 17 (19.5%) |
| I would not have been able to quit without the program | 34 (39.0 %) | 11 (12.6 %) | 23 (26.4%) | 42 (48.3%) | 6  (6.9%) | 5  (5.7%) | 11 (12.6%) |
| I stopped using it by the end of the program | 28 (32.1%) | 11 (12.6%) | 17 (19.5%) | 32 (36.8%) | 21 (24.1%) | 6  (6.9%) | 27 (31.0%) |
| information was easy to understand | 70 (80.5 %) | 26 (29.9 %) | 44 (50.6%) | 14 (16.1%) | 2  (2.3 %) | 1  (1.1%) | 3  (3.4%) |
| information was useful for me | 70 (80.5%) | 21 (24.1%) | 49 (56.3%) | 13 (14.9%) | 3  (3.4%) | 1  (1.1%) | 4  (4.5 %) |
| The information mentioned about what I was experiencing and feeling | 57 (65.5%) | 17 (19.5%) | 40 (46.0%) | 28 (32.2%) | 0 | 2  (2.3%) | 2  (2.3 %) |
| I received too much information | 29 (33.3%) | 10 (11.5%) | 19 (21.8%) | 42 (48.3 %) | 16 (18.4%) | 0 | 16 (18.4%) |
|  |  | **Almost never** |  | ≥**1 time a week** |  | **everyday** |  |
| Frequency of using app |  | 16 (18.4 %) |  | 49 (56.3%) |  | 22 (25.3%) |  |

^a^CBT: cognitive behavioral therapy.

^b^Data are n (%), n: number.

^c^n: number.
